# Seeing ‘Where’ through the Ears: Effects of Learning-by-Doing and Long-Term Sensory Deprivation on Localization Based on Image-to-Sound Substitution

**DOI:** 10.1371/journal.pone.0001840

**Published:** 2008-03-26

**Authors:** Michael J. Proulx, Petra Stoerig, Eva Ludowig, Inna Knoll

**Affiliations:** Institute of Experimental Psychology, Heinrich-Heine-University Düsseldorf, Düsseldorf, Germany; University of Oxford, United Kingdom

## Abstract

**Background:**

Sensory substitution devices for the blind translate inaccessible visual information into a format that intact sensory pathways can process. We here tested image-to-sound conversion-based localization of visual stimuli (LEDs and objects) in 13 blindfolded participants.

**Methods and Findings:**

Subjects were assigned to different roles as a function of two variables: visual deprivation (blindfolded continuously (Bc) for 24 hours per day for 21 days; blindfolded for the tests only (Bt)) and system use (system not used (Sn); system used for tests only (St); system used continuously for 21 days (Sc)). The effect of learning-by-doing was assessed by comparing the performance of eight subjects (BtSt) who only used the mobile substitution device for the tests, to that of three subjects who, in addition, practiced with it for four hours daily in their normal life (BtSc and BcSc); two subjects who did not use the device at all (BtSn and BcSn) allowed assessment of its use in the tasks we employed. The impact of long-term sensory deprivation was investigated by blindfolding three of those participants throughout the three week-long experiment (BcSn, BcSn/c, and BcSc); the other ten subjects were only blindfolded during the tests (BtSn, BtSc, and the eight BtSt subjects). Expectedly, the two subjects who never used the substitution device, while fast in finding the targets, had chance accuracy, whereas subjects who used the device were markedly slower, but showed much better accuracy which improved significantly across our four testing sessions. The three subjects who freely used the device daily as well as during tests were faster and more accurate than those who used it during tests only; however, long-term blindfolding did not notably influence performance.

**Conclusions:**

Together, the results demonstrate that the device allowed blindfolded subjects to increasingly know where something was by listening, and indicate that practice in naturalistic conditions effectively improved “visual” localization performance.

## Introduction

Vision is “to know what is where by looking” (p. 3 [1]). This definition is intuitively appealing because it describes two central purposes of vision: object recognition and localization. The blind have to rely largely on auditory and tactile information for finding and identifying objects. Sensory substitution aims at supplementing the available aids (such as the cane, echolocation devices, and Braille script) by converting visual information into a tactile or auditory format (see [2] for a general review). The resultant tactile arrays or sound patterns inform a blind person whether, where, and what silent objects fall within the field of view of the camera whose input they represent. Although substitution devices are capable of providing both what and where information, most studies have explored the potential of sensory substitution for stimulus discrimination, often using very simple stimuli [3]–[7]. As only one study [8] has had their subjects localize and explore real objects with a hand-held camera whose signals were converted into sound patterns, localization performance has hardly been addressed (however see [9] for a study of the estimation of distance in depth for simple stimuli using a joystick and computer interface and [10] for a study of spatial navigation). Moreover, studies to date have exclusively employed in-session learning to show that training improves discrimination performance over sessions in blind participants as well as in subjects blindfolded during training [3]–[8].

**Figure 1:**
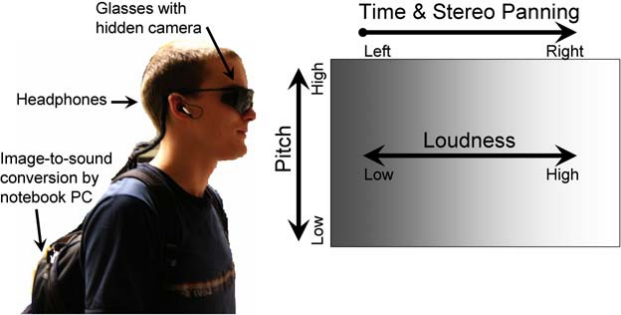
An illustration of the sensory substitution device and its conversion principles. A) The vOICe program is installed on the notebook computer in the backpack. The camera is hidden in the glasses and the earphones provide the result of the image-to-sound conversion. B) Conversion principles for The vOICe.

The present study used an image-to-sound conversion program, The vOICe [11], to examine the perceptual learning of manual localization based on the sounds generated by translating the images from a video camera hidden in sunglasses (see Figure 1). The use of a head-mounted rather than a handheld camera (cf. [8]) requires a different coordinate system and different sensory-motor contingencies for using the camera to allow one to grasp objects. Localization was assessed in three experiments. In the first, the participants had to manually indicate the location of a lit LED in a horizontal array of 18 possible target locations. The second experiment examined whether the learning would transfer to a more challenging LED task where there were 164 possible target locations. In the third experiment objects that were placed singly on a large table had to be located and grasped. On the basis of subjects' grasping precision we were also able to consider the ability of participants to take account of features of the object, such as its size.

**Figure 2:**
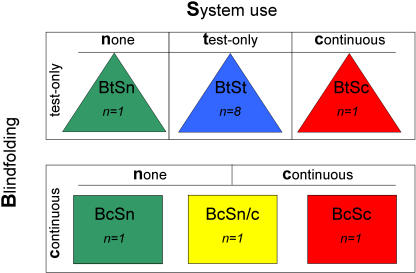
Assignment of subjects to the experimental conditions. Subject assignment to the experimental conditions was created by partially crossing blindfolding with use of the sensory substitution system. Bc = Blindfolding continuous (for 21 days); Bt = Blindfolding test-only; Sc = System use continuous (in daily life and for all tests); St = System test-only (for all test but not in daily life); Sn = no System used (for the tests or in daily life). Note that subject BcSn/c was a cross between BcSc and BcSn because he was blindfolded continuously for 21 days, did not have the system for the first 11 days, but did use it in daily life and the tests for the final 10 days. The colors represent the extent of system use in Figures 3 to [Fig pone-0001840-g004] 5, the shapes the extent of blindfolding in Figures 3 and 5.

Unlike all published studies on sensory substitution that employed structured in-session learning to show that training improves the performance of both blind and blindfolded subjects, we here compared within-session learning to a learning-by-doing approach in naturalistic conditions (see Figure 2 for the conditions that defined our subjects). This naturalistic learning was investigated by providing the mobile substitution system to three subjects for use in their daily lives. Two of these subjects had the system continuously for 21 days (BtSc and BcSc); the third had it for the final 10 days only (BcSn/c), and therefore provided a within-subject assessment of the effects of daily practice on performance. Eight subjects used the system during the tests only (BtSt); this group essentially replicated the normal subject group in other studies of sensory substitution (e.g. [8]) who only benefit from in-session practice. Two final subjects did not use the system at all (BtSn and BcSn). The three groups allowed us to assess the effect of using the system during the tests, and to compare in-session to in-session plus naturalistic learning.

Finally, we studied the impact of sensory deprivation on the learning of sensory substitution by blindfolding three of the thirteen subjects (BcSn, BcSn/c, and BcSc) for the entirety of 21 days (24 hours per day), the longest, non-clinical period of visual deprivation in the literature (for a previously long duration of 5 days, see [12]). The ten others were only blindfolded during the laboratory tasks, similar to previous research (BtSn, BtSc, and the eight BtSt subjects). Long-term rather than test-only blindfolding was used in three participants because it may enhance perceptual learning both for the remaining modalities that have to compensate for the visual deprivation, and by rendering subjects more dependent on the system (e.g., [13]–[14]).

Taken together, the study has three contributions to the literature on sensory substitution: First it focused on localization (see also [8], [9], [10]); second, it compared in-session to naturalistic learning by providing some subjects with the equipment necessary for practicing with the device in their daily lives; and third it examined the effects of long-term sensory deprivation on the learning of sensory substitution.

## Results

### Experiment 1: Horizontally Located Light Source

#### System use (continuous, test-only or not at all)

**Figure 3:**
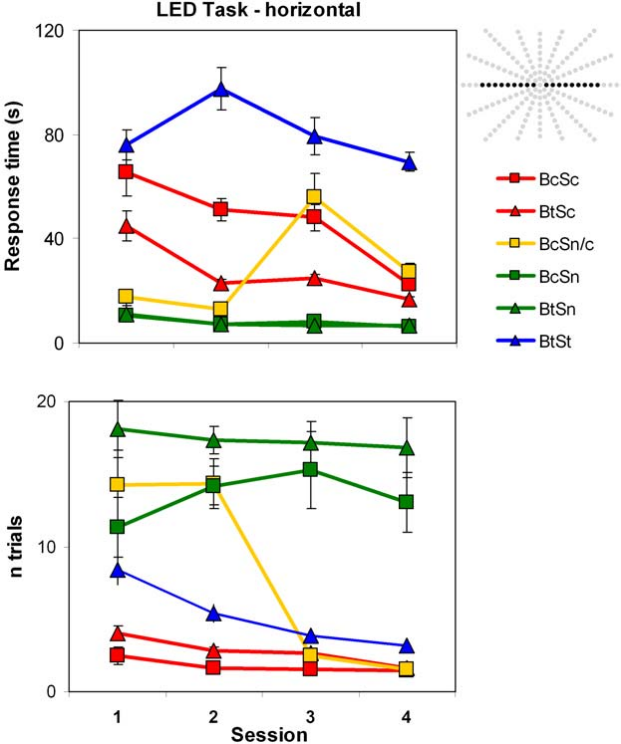
The horizontal LED task results from Experiment 1. Top) Response times as a function of session. The inserted array shows the potential target LEDs (black) in the perimeter. The subjects knew that these were confined to the horizontal meridian. Bottom) Accuracy is plotted as a function of the number of LEDs pushed on a given trial until hitting the target (“1” is perfect performance). All error bars denote standard error of the means in this and all figures.

Sn subjects (who wore no device for the tests; green shapes in Figure 3) were much faster than those who used the system; however, accuracy was expectedly at chance level. There were 18 LEDs that could potentially be the target, and the subjects without the system had to press almost as many, on average, before hitting the target LED (mean 15 LEDs per trial). There was no change in accuracy (*r* = 0.13, *p* = 0.36, *p*_rep_ = 0.60, *d* = 0.26) over the sessions. In contrast, response times improved strongly as a function of session number (*r* = −0.70, *p* = 0.012, *p*_rep_ = 0.95, *d* = 1.96) for the Sn subjects, presumably because they learned to hit as many LEDs as they could, as fast as they could, and increasingly used both hands for the task.

The data of the BtSt subjects who only used the device for the testing session are plotted with blue triangles in Figure 3. This group took much longer to find the target LED than the Sn subjects, and their response times did not decrease across sessions (*r* = −0.15, *p* = 0.211, *p*_rep_ = 0.71, *d* = 0.30). In further contrast to the Sn subjects, their accuracy improved from session to session (mean n trials 8.4 in session 1 versus 3.2 in session 4; *r* = −0.43, *p* = 0.007, *p*_rep_ = 0.96, *d* = 0.95). The Sc subjects who used the device during the tests as well as in daily life were faster (mean RT 38 s; red shapes in Figure 3) than the St (80.5 s), but slower than the Sn subjects s (mean 8 s). These Sc subjects showed excellent accuracy which improved significantly (mean n trials 3.3 in session 1 versus 1.5 in session 4; *r* = −0.71, *p = *0.011, *p*_rep_ = 0.95, *d* = 2.0) along with search time (*r* = −0.65, *p = *0.022, *p*_rep_ = 0.92, *d* = 1.71) across sessions. Note that they became almost as fast (mean 22 s) in Session 4 as an Sn subject who systematically pressed all LEDs in Session 1 (subject BcSn/c, mean 17.5 s). That Sc subjects improved both in accuracy and speed suggests that their daily use of the system outside of the testing sessions, and the many opportunities it provided for learning to adjust their image-to-sound guided behavior to the camera's field of view, contributed to their significant improvement on both counts in this laboratory task.

#### Blindfolding (continuous or test-only)

We compared the Bc and Bt subjects to investigate whether continued visual deprivation affected performance in this task. Response times did not decrease substantially for either group (Bc, *r* = −0.13, *p* = 0.343, *p*_rep_ = 0.61, *d* = 0.26; Bt, *r* = −0.12, *p* = 0.226, *p*_rep_ = 0.70, *d* = 0.24), but, as seen in Figure 3, was lower for the Bc subjects throughout. Although accuracy for both the Bt and Bc groups improved similarly from the first (9.3 trials-to-hit for Bc, 8.9 for Bt) to the last (5.4 for Bc, 4.4 for Bt) session, it improved consistently only for the Bt (*r* = −0.30, *p* = 0.03, *p*_rep_ = 0.91, *d* = 0.63), but not the Bc subjects (*r* = −0.29, *p* = 0.18, *p*_rep_ = 0.74, *d* = 0.61).

#### Continuous system use and blindfolding

Both Sc subjects performed well and exhibited perceptual learning. The continuously blindfolded subject BcSc initially had higher accuracy than BtSc; both exhibited improvement (BcSc: *r* = −0.89, *p* = 0.058, *p*_rep_ = 0.87, *d* = 3.90), though BtSc had a higher correlation between accuracy and testing session (BtSc *r* = −0.97, *p* = 0.017, *p*_rep_ = 0.93, *d* = 7.98). Conversely, BtSc had faster RTs to begin with, and though she showed some improvement (*r* = −0.88, *p* = 0.063, *p*_rep_ = 0.86, *d* = 3.71), BcSc had a higher correlation (*r* = −0.95, *p* = 0.024, *p*_rep_ = 0.92, *d* = 6.08) and better accuracy throughout. Unsurprisingly, in the first two tests where he performed without the system, BcSn/c was fast and inaccurate. When he first used a system in session 3, his search time as well as his accuracy increased dramatically. In session 4, the second session he performed while using the device, his search times already decreased by half, and his localization was as precise as that of the two other Sc subjects who had performed three prior sessions with the system.

### Experiment 2: Hexagonally Located Light Source

In Experiment 1, all subjects knew that the target LED would be located on the horizontal row of 18 LEDs. To investigate whether the learning would transfer to a task in which subjects did not know where in the perimeter the targets might be, and had to consider all 164 LEDs as possible targets, we used a different arrangement at the end of the 4^th^ and final session. The subjects were not informed that only six LEDs were actually used, or that each served as target twice.

**Figure 4 pone-0001840-g004:**
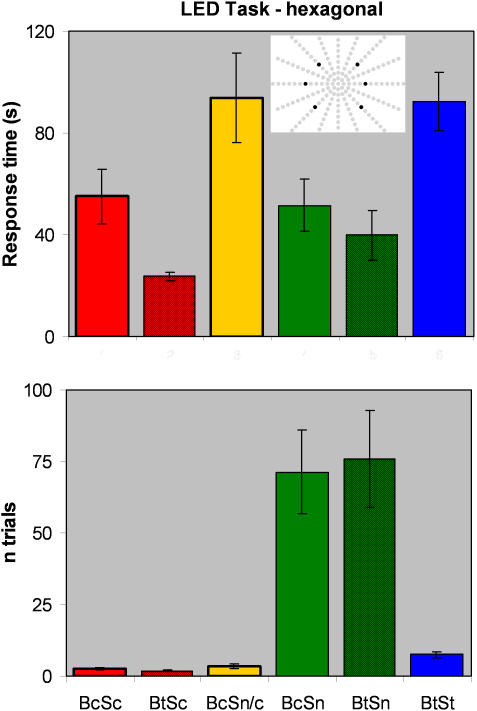
The hexagonal LED task results from Experiment 2. Each of the six hexagonally arranged LEDs shown in the insert served twice as target, but as subjects were not informed about this array, they had to consider all 164 LEDs as possible targets. Mean response time (top) and number of trials to hit the target (bottom) as a function of the subject's condition (visual deprivation and system use for each subject).

Figure 4 depicts the results. The bottom panel shows the mean number of LEDs pressed up to and including the target.

#### System use (continuous, test-only or not at all)

The Sn subjects needed many more trials to hit the target than in the preceding tests (mean trials to hit 73.6), and their response times were relatively fast (mean 45.8 s). As in the ‘horizontal’ task, the BtSt subjects took longer to find the targets than the Sn subjects (BtSt, mean 92.3 s; Sn, mean 45.8 s; *t*(82) = 3.45, *p = *0.0004, *p*_rep_ = 0.99, *d* = 0.76; the significant *p* value for this and all tests is 0.01 when Bonferroni corrected for multiple comparisons), but had much better accuracy (mean 7.4 trials compared to 73.6 trials for Sn; t(23) = 5.97, p<0.0001, p_rep_ = 1.0, d = −2.5). The Sc subjects performed even better than the BtSt subjects, pressing no more than one or two LEDs adjacent to the target on almost all of the trials (mean trials to hit 2.5, median 2, mode 1; *t*(70) = 4.6, *p*<0.0001, *p*_rep_ = 1.0, *d* = 1.1. Furthermore, their response times were statistically similar to those of Sn subjects (with device, mean 57.5 s; without device, mean 45.8 s; *t*(58) = 1.1, *p = *0.13, *p*_rep_ = 0.77, *d* = 0.28), and considerably faster than those of the BtSt subjects (92.3 s versus 57.5 s; *t*(94) = 2.5, *p = *0.008, *p*_rep_ = 0.96, *d* = 0.51). The increased difficulty of this task confirms the superior localization performance the Sc subjects had obtained after using the system in daily life for 21 or even just 10 days.

#### Blindfolding (continuous or test-only)

The effect of visual deprivation on the hexagonal LED task, independent of system-use, was mixed. The Bt subjects who were seeing in daily life found the target faster than the Bc subjects who were blindfolded continuously (32 s for Bt versus 67 s for Bc; *t*(55) = 3.6, *p = *0.0003, *p*_rep_ = 0.99, *d* = 0.98). The accuracy of the Bt and Bc subjects was not statistically different (*t*(41) = 1.68, *p = *0.17, *p*_rep_ = 0.75, *d* = 0.30). However, the numerical trend indicated that the Bc subjects had better accuracy than the Bt subjects (mean n trials to find the target was 26 for Bc versus 39 for Bt).

#### Continuous system use and blindfolding

The most interesting finding of this experiment, in comparison to Experiment 1, is that the two Sc subjects that used the device daily not only had greater accuracy than the Sn subjects but also had search times that were as fast as or faster than the Sn subjects. The difference in accuracy is clear in Figure 4. Beyond the previous analyses that demonstrated that the response times were not statistically distinguishable for the Sn versus the Sc subjects, it is also interesting to note that subject BtSc was faster than the fastest Sn subject (24 s for BtSc versus 40 s for BtSn; *t*(12) = 1.65, *p = *0.062, *p*_rep_ = 0.86, *d* = 0.95).

### Experiment 3: Finding Objects on a Table

Whereas the first two experiments focused solely on “where” information, the final one also considered object features pertaining to “what” information, such as an object's size and shape. Different ordinary objects were used on each trial, and subjects had to localize and grasp them. This allowed us to analyze search times as well as how directly the subjects reached for the objects and how appropriate their hand grip was for the object.

#### System use (continuous, test-only or not at all) and search time

**Figure 5:**
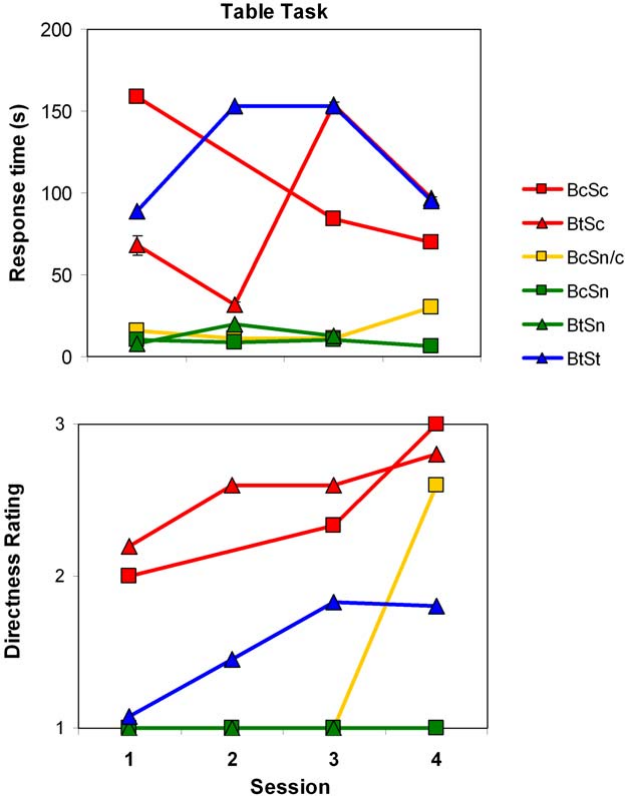
The table task results from Experiment 3. Top) Mean response time as a function of session. Bottom) Rating of grasp precision as a function of session (1 = indirect; 2 = relatively direct; 3 = direct). The photograph at the top right shows a directed grasp of the target object by a subject (BcSc) using the substitution device.

Figure 5 shows the search time data from the third experiment, where subjects had to find various everyday objects, presented one at a time on a table. They exhibited high variability and, for the Sc and Sn subjects, search times correlated weakly with session number (Sc, *r* = −0.26, *p* = 0.26, *p*_rep_ = 0.68, *d* = 0.54; Sn, *r* = −0.29, *p* = 0.21, *p*_rep_ = 0.71, *d* = 0.61; top panel of Figure 5). The BtSt subjects showed no improvement in search time (*r* = 0.026, *p* = 0.44, *p*_rep_ = 0.54, *d* = 0.05). As we had to use different tables for the BtSt subjects and the other participants, absolute search times cannot be compared. Note, however, that the search times for the BtSt and two Sc subjects are very similar on average, suggesting that the difference in table size did not have much impact on the search time results.

#### Blindfolding (continuous or test-only) and search time

Variability was also high when considering the Bc versus the Bt subjects, and again there was no clear improvement in search time across session numbers (*p*>0.25, *p*_rep_<0.70). A comparison of the subjects who used no device (BcSn, BtSn, and BcSn/c during the first three sessions) reveals no advantage of continuous blindfolding (in the absence of using the system) in this task.

#### System use (continuous, test-only or not at all) and directed grasping

We analyzed the grasping behavior of subjects to determine how much the subjects' grasping took account of the objects' position, size and orientation (see Figure 5 bottom panel). The Sn subjects had ratings that corresponded with indirect grasping across all sessions. This reflects their strategy: to slide their hands across the entire table, stepping sideways to reach all edges until an object was discovered tactually. The St subjects started almost as poorly, but increased their directness of grasping up to session 3 where it reached a plateau (*r* = 0.93, *p* = .034, *p*_rep_ = 0.90, *d* = 5.06). Finally, the Sc subjects clearly improved directness in their grasping of the object (*r* = 0.84, *p* = .005, *p*_rep_ = 0.97, *d* = 3.10).

#### Blindfolding (continuous or test-only) and directed grasping

There was an improvement in directed grasping across test sessions for the Bt (*r* = 0.27, *p* = .048, *p*_rep_ = 0.88, *d* = 0.56) and Bc subjects (*r* = 0.45, *p* = .081, *p*_rep_ = 0.84, *d* = 1.01). Note that there were more Bt subjects than Bc (11 versus 2) which resulted in the lower correlation having a lower, and therefore statistically significant, *p* value. The Bc subjects, however, had the higher correlation and effect size, suggesting that continuous blindfolding had a positive effect on grasping.

#### Blindfolding and system use (single subject analyses)

BtSc performed very precisely from the first session, and BcSc grasped all objects directly in the last one (see Figure 5 for an example photograph). BcSn/c performed only a single session with the system. Nevertheless, he also grasped three objects directly, and two relatively direct, suggesting that the practice he had with the system in his daily life, plus perhaps having to adapt to daily life with a blindfold in the absence of the system for the first half of the period, played a substantial role in improving his search strategy as well as his reaching (see supporting online material for video examples of subject BcSn/c in Movie S1 and BcSc in Movie S2 and Movie S3). BcSn/c also had search times that were faster than BcSc in the final session even though he had less experience with the system overall (30 s for BcSn/c versus 70 s for BcSc; *t*(4) = 1.66, *p = *0.085, *p*_rep_ = 0.83, *d* = 1.67). Although this difference is not statistically significant due to low power and might arise because of individual differences between the subjects, there is also a possibility that one who has adapted to sensory deprivation in the absence of using a substitution device (as the blind have) may be able to learn to use such a device more quickly and with better performance.

## Discussion

Here we examined the impact of naturalistic learning and sensory deprivation on the perceptual learning of object localization via image-to-sound substitution. As noted in the Introduction, this study has three primary contributions to the literature on sensory substitution: 1) it focused primarily on the less studied localization (see also [8], [9], [10]); 2) it employed a naturalistic, learning-by-doing approach in addition to the in-session practice that is normally employed, and 3) it featured the longest non-clinical blindfolding of subjects in addition to the standard in-session-only blindfolding.

1. Localization performance, hitherto only tested with a hand-held camera [8] or a joystick [9], [10], which might also impede one's ability to make free use of one's hands, improved in both Experiments 1 and 3 in subjects who used the system. No such improvement occurred in the Sn subjects who had chance accuracy throughout. These subjects were much faster than those who used the system, at least as long as the target array was limited. The Sn subjects decreased their search times consistently over sessions in Experiment 1 due to employing more effective strategies, such as using two hands to touch the LEDs more quickly. Search times also decreased in the initially much slower St and Sc subjects who were particularly challenged by having to adjust to the smaller field of view of the camera, and to the sweep time of the conversion program, as both required appropriate adaptation of head and, especially in Experiment 3, body movements. Nevertheless, Sc and St subjects had to press fewer LEDs before hitting the target in Experiments 1 and 2, and unlike the Sn subjects, also improved the precision of their grasping of objects in Experiment 3. As our targets were clearly defined – only the target LED was lit, and only one object was positioned on the table at a time – we cannot conclude that a more difficult task, such as finding a particular object among distracters, will be learned as effectively. However, the improvements we observed in the hand posture during reaching gives reason for cautious optimism.

2. Previous studies only focused on laboratory practice. Our BtSt group essentially replicates this approach, and, as in other reports [3]–[10], revealed statistically significant improvements across four sessions. However, the Sc subjects who used the substitution system immersively in their daily life had superior performance to that of the St, in-laboratory, users of the device in all three experiments. Together, the results suggest that the additional daily practice and the opportunities for learning-by-doing in naturalistic conditions it afforded effectively improved performance on the localization tasks we presented. Future research will have to show whether the naturalistic-learning conditions or the additional hours of practice account for the Sc subjects' enhanced performance.

3. Continuous, rather than test-only visual deprivation might lead to greater perceptual learning of localization than just using the system alone. Although our results are not as straightforward in this respect as a previous study [13] which found that Braille learning profited markedly from five days of continuous blindfolding, the results from our third experiment suggest that the combination of immersive use and extended sensory deprivation may be particularly effective. Subject BcSn/c, who spent the first half of the experiment blindfolded but only had the device for the second half, exhibited very rapid learning and even had superior localization performance in Experiment 3 over that of BcSc who had the system for the duration of the experiment. Although any conclusion we could draw is tempered by the small number of our Bc subjects, the blind for whom the system is designed, may thus progress faster.

The learning necessary to use the device involves not only perceptually matching the auditory input to a representation of an object or scene that is derived from vision or touch, but other types of learning as well. Subjects must learn to remap egocentric space to match the camera's viewpoint, angle, and field of view. They must adjust their head and body movements to these properties, so as not to miss a possibly vital part of the scene. In addition, they must learn to adapt their movements to the sweep rate used by the system which only provides a snapshot of the scene every one or two seconds; in fact, many subjects made fast, large head movements in the early testing sessions and noticeably more deliberate, slower, and smaller head movements later in the study. Future studies that try to determine the most effective training protocols will have to address these different types of learning. Moreover, as the adult brain that has been subject to actual, peripheral blindness is very likely different from one that has been exposed to short-term blindfolding [15], studies with blind subjects are important for understanding the learning that accompanies sensory substitution and for improving such systems for use by the blind.

In summary, the adult auditory system can learn to localize targets based on an image-to-sound conversion system, and immersive practice holds hope for providing the perceptual learning required to localize things quickly and accurately. Most of our results speak to the question of object localization. However, the increased directness of the grasping in Experiment 3 suggests that the subjects also gained general knowledge of the objects' size and shape. By allowing blindfolded subjects to increasingly hear where silent objects are, the system provides knowledge about what is where by listening.

## Materials and Methods

### Participants

Having obtained approval from the University's Ethics Committee, we tested 13 sighted subjects (see Figure 2) with informed verbal consent. All were informed about the principles of image-to-sound conversion, and subjects who used the mobile substitution system only during tests (St, System-test-only) as well as those who additionally used it in daily life (Sc, System-continuously) were instructed in its use. None had prior experience with sensory substitution. As the utility of the substitution system was difficult to judge on solely its own merits, a comparison group (two additional subjects who never used the system (Sn, System-none)) was included. Of the ten subjects who were blindfolded only for the testing sessions (Bt subjects, Blindfolded-test-only, five female, age 23–46 yrs), nine were students, and one was associated with the laboratory. The three subjects who were visually-deprived during the entire experiment (Bc subjects, Blindfolded-continuously, one female, age 25–39 yrs) were selected from among a large number of volunteers; they had to be intrinsically motivated (for instance by having a blind relative) and had to be living with someone who agreed (in writing) to look after them during the experimental period. On day 1, one Bt and one Bc subject were equipped with a substitution device (see Figure 1); subject BcSn/c received his system on day 11 to use for the second half of the period, and thus served as an intra-subject control. All Sc subjects were asked to use the system daily for at least four hours. Compliance was very good, as established through close contact with the experimenters and the daily reports subjects provided. Sc and Sn subjects participated in further experiments during this period [16], and received financial compensation. All subjects were blindfolded during the laboratory tests where Sc and St subjects used a substitution device.

### Sensory Substitution Device

Our substitution system consisted of a small video camera hidden in sunglasses (Mace Security Products Eyeglasses Camera ST-137W) and a notebook computer (IBM ThinkPad) that received the camera's digitalized signals, and converted them into sound patterns played to the subject by means of stereo headphones (Figure 1). The camera provided a field-of-view that subtended approximately 39° by 31° of visual angle. The vOICe program uses three major conversion dimensions: 1) laterality is coded by stereo panning and the time provided by the left-to-right scanning transformation of each image (the precision of the time scanning is fixed, and users can choose the rate of scanning to occur every one or two seconds), so that the sound pertaining to an object on the right of the image will be heard late in the scan and predominantly through the right ear; 2) elevation is coded by frequency, so that down is represented by low frequencies and up by high frequencies (an exponential distribution from 500 Hz to 5000 Hz); 3) pixel brightness is coded by loudness (Figure 1b). A single bright object on an otherwise dark surface will thus generate a sound pattern whose loudness reflects its brightness, whose duration and frequency spectrum represent its size, and whose frequency modulations represent its shape (see supporting online material, Movie S4 for an example image converted into sound).

### Statistical analyses

The study primarily focused on the perceptual learning of sensory substitution. We were therefore interested in how the variables impacted the performance of the subjects over time. A negative correlation (Pearson's *r*) of search times and errors with testing session was expected if performance improved over the three weeks of the study. For each experiment we first analyzed the data in terms of the manipulation of system use, then the manipulation of blindfolding, and finally we looked at individual subjects to consider the interaction between blindfolding and system use.

All data analyses were conducted using the *p*_rep_ statistic [17]. Note that we also provide the standard *p* statistic for comparison and standard interpretation. We include the *p*_rep_ statistic because it overcomes a primary problem with null hypothesis statistical tests (i.e., the inability to accept or reject the null hypothesis), and it also provides a measure of the probability of replicability that is of primary importance in all research, but especially when considering small-*n* research such as that presented here. Thus, data can be interpreted with the following guideline: the higher the *p*_rep_ statistic, the greater the likelihood that the results will be replicable. The values of *p*_rep_ are directly proportional to *p* values, however: Values of *p*_rep_ greater than 0.9 are equal to *p* values significant at an alpha level of 0.05. We also provide effect sizes (using Cohen's *d*) for an additional evaluation of our results [18].

#### Experiment 1: Horizontally Located Light Source

A semi-cylindrical perimeter fitted with touch-sensitive red LED buttons was used for this task (see inset depiction in Figure 3). With a diameter of 90 cm and a radius of 45 cm, it formed a semicircle in the horizontal plane, with 165 LED buttons arranged in a star-like pattern. All subjects were blindfolded during testing, and first moved their hands over the perimeter's inner surface to acquaint themselves with the layout of the LEDs. Seated centrally, they started each trial by pressing a start button located on the table in front of them. This activated one of the LEDs as well as a small loudspeaker at the top-center of the perimeter that began a buzzing sound (500 Hz, adjustable volume) which continued until the subject pressed the appropriate – illuminated – LED button. This response extinguished both the light and the sound, informing the subjects that they had found the target. The subjects that used the vOICe device (St and Sc) could still hear the sound that announced the start and continuation of a new trial, and none reported any difficulty hearing the output of the device as a result of the external steady tone. Subjects were informed about this procedure, and also knew that only the 18 LEDs along the horizontal row would be used. Ten subjects used the audiovisual substitution system for the tests (Bc and Bt), one performed it first twice without, then twice with the system (BcSn/c), and two performed it without the device (BtSn and BcSn). All subjects using a device started with a sweep rate of one image per two seconds, but were free to accelerate the sweep rate to one image/s after one to four series. Whereas the subjects with the device were instructed to localize the LED before attempting to press it, the subjects without the device simply pressed as many LED buttons as necessary until the correct one was reached. A PC recorded each LED button pressed during the search, and measured the time from the onset of the light stimulus to the correct response.

Each LED subtended 1.9° at a viewing distance of approximately 45 cm. The experiment used 18 LEDs. They were distributed evenly along the horizontal meridian, with a center-to-center distance of 6.3°; only the distance between the two most central target LEDs was twice as large, because the centralmost LED that normally serves as continuously-lit fixation spot was covered with black felt. Each LED had a luminance of ∼8 cd/m^2^, and was illuminated once per series. Ambient luminance was low (0.15–0.5 cd/m^2^) to increase target salience for the subjects who used the device. One or two series were given per session; only BtSc enthusiastically performed five in the second session.

#### Experiment 2: Hexagonally Located Light Source

The apparatus and procedure for Experiment 2 was similar to that used for Experiment 1, except for the changes noted below.

##### Participants

The two Sn, the three Sc, and five St subjects participated.

##### Apparatus and Procedure

As illustrated in the inset depiction in Figure 4, two active LEDs were in the upper quadrants, two on the horizontal, and two in the lower quadrants. All subjects used a sweep rate of 1 image/s.

#### Experiment 3: Finding Objects on a Table

For each of the five trials per session, a single object was placed on a large table completely covered with black felt-like cloth to provide enhanced contrast to aid the subjects in their search for the objects placed on it. Table size was 2.6×1.4 m for the BtSt subjects who were tested in Düsseldorf, and 2 by 1.1 m for the other five subjects who were tested at the Jülich Research Center where parts of the experiments were conducted. Object position was varied pseudo-randomly, and care was taken to mask any auditory cues to the object's position that could result from hearing the experimenter's footsteps or the placement itself. The subject was asked to try and find the object, and started searching while standing by the long side of the table. Five different objects that varied both in size (e.g. a pen, a CD, a trainer, a large box) and in contrast (a white shirt rolled into a ball, a gray plush mouse) to the cloth were used for each testing period, with new objects selected for each session. The subjects did not know the identity of the objects until they found and grasped them.

As in Experiment 1, all subjects were blindfolded. The Sc and St subjects used the device during the tests; the one who had the system for 10 days at the end of the experimental period (BcSn/c) performed it with the device in only the final, fourth testing session. The two Sn subjects performed blindly throughout, but, unlike those using a system, were allowed to slide their hands across the surface of the table to find the objects; as in the previous experiments, Sc and St subjects were asked to use the sound patterns for this purpose. Trials were recorded by digital video camera (Sony Digital Handycam), to time the searches by using the camera's digital clock and to assess the precision of the grasping movements. Note that the data for the second session by BcSc is missing because the camera did not record that session.

Grasping for the objects was coded as either: indirect (coded as 1), relatively direct (2), or direct (3) by two raters. Sliding the hands in a sweeping manner, rather than towards an object, was coded as 1. Reaching that was directed in the general vicinity of the object, but was followed by a tactual search, was coded as 2. Direct grasping (3) was attested when the reaching movement was directed at the object, errors were confined to those of depth (over- or under-reaching grasps), and the hand-posture was largely appropriate to the size, shape and orientation of the object. The average across the five objects tested in each session was taken, and then subjected to the analyses and figural depiction described in the Results section. A comparison between the primary and second rater resulted in a high interrater reliability (*r* = .966; full agreement on 96% of the coded trials).

## Supporting Information

Movie S1 Here the subject that was continuously blindfolded and had the sensory substitution device for only the final session of Experiment 3 is shown directly grasping an object.
(2.39 MB MOV)

Movie S2 Here the subject that was continuously blindfolded and had the sensory substitution device continuously is shown directly grasping an object.
(1.15 MB MPG)

Movie S3 Here the subject that was continuously blindfolded and had the sensory substitution device continuously is shown directly grasping another object.
(0.46 MB MPG)

Movie S4 Here is a video demonstrating the conversion principles in Figure 1. Here an image of three squares is transformed into sound with a sweep rate of two seconds.
(0.05 MB MPG)
